# Gold 2023: Highlights for primary care

**DOI:** 10.1038/s41533-023-00349-4

**Published:** 2023-07-31

**Authors:** Alvar Agustí, Antoni Sisó-Almirall, Miguel Roman, Claus F. Vogelmeier, On behalf of the members of the Scientific Committee of GOLD (Appendix), Antonio Anzueto, Peter Barnes, Jean Bourbeau, Bartolome R. Celli, Gerard J. Criner, David Halpin, MeiLan K. Han, Fernando J. Martinez, Maria Montes de Oca, Kevin Mortimer, Alberto Papi, Ian Pavord, Nicolas Roche, Sundeep Salvi, Don D. Sin, Dave Singh, Robert Stockley, M. Victorina López Varela, Jadwiga A. Wedzicha

**Affiliations:** 1 Cátedra Salud Respiratoria, Univ. Barcelona, Hospital Clinic, IDIBAPS and CIBERES, Barcelona, Spain; 2 Consorci d’Atenció Primària de Salut Barcelona Esquerre (CAPSBE). Grup de Recerca Transversal en Atenció Primària (IDIBAPS). Departament de Medicina, Universitat de Barcelona, Barcelona, Spain; 3 Univ. Islas Baleares, Instituto de Investigación Sanitaria de las Islas Baleares (IdISBa), centro de salud Son Pisa Palma de Mallorca, Palma de Mallorca, Spain; 4 Department of Medicine, Pulmonary and Critical Care Medicine, University Medical Center Giessen and Marburg, Philipps-University, German Center for Lung Research (DZL), Marburg, Germany; 5 South Texas Veterans Health Care System University of Texas, Health San Antonio, San Antonio, TX USA; 6 National Heart & Lung Institute Imperial College London, London, UK; 7 McGill University Health Centre McGill University Montreal, Montreal, Canada; 8 Brigham and Women’s Hospital, Harvard Medical School, Boston, MA USA; 9 Lewis Katz School of Medicine at Temple University, Philadelphia, PA USA; 10 University of Exeter Medical School College of Medicine and Health University of Exeter, Exeter Devon, UK; 11 University of Michigan, Ann Arbor, MI USA; 12 Weill Cornell Medical Center/ New York-Presbyterian Hospital, New York, NY USA; 13 Hospital Universitario de Caracas Universidad Central de Venezuela Centro Médico de Caracas, Caracas, Venezuela; 14 Liverpool University Hospitals NHS Foundation Trust, Liverpool, UK; 15 National Heart and Lung Institute, Imperial College, London, London, UK; 16 School of Clinical Medicine, College of Health Sciences, University of Kwazulu-Natal, Kwazulu-Natal, South Africa; 17 University of Ferrara, Ferrara, Italy; 18 Respiratory Medicine Unit and Oxford Respiratory NIHR Biomedical Research Centre, Nuffield Department of Medicine University of Oxford, Oxford, UK; 19 Pneumologie, Hôpital Cochin AP-HP.Centre, Université Paris, Paris, France; 20 Pulmocare Research and Education (PURE) Foundation, Pune, India; 21 St. Paul’s Hospital University of British Columbia, Vancouver, Canada; 22 University of Manchester, Manchester, UK; 23 University Hospital, Birmingham, UK; 24 Universidad de la República. Hospital Maciel Montevideo, Montevideo, Uruguay

**Keywords:** Diseases, Chronic obstructive pulmonary disease

## Introduction

The Global Initiative for Chronic Obstructive Lung Disease (GOLD) has issued its 2023 annual report^1^. Compared with former versions, it has been significantly updated. Here, we summarize the most relevant changes for a Primary Care audience. The complete document can be downloaded for free from the GOLD web page (www.goldcopd.org), together with a “pocket guide” and a “teaching slide set”.

## New definition

GOLD 2023 defines COPD as a heterogeneous lung condition characterized by chronic respiratory symptoms (dyspnoea, cough, expectoration and/or exacerbations) due to abnormalities of the airways (bronchitis, bronchiolitis) and/or alveoli (emphysema) that cause persistent, often progressive, airflow obstruction (FEV1/FVC < 0.7)^1^. This definition aims at: (1) recognizing that COPD is heterogeneous; and (2) describing explicitly what are the main structural, functional, and clinical manifestations of the disease.

## Causes and risk factors

Traditionally, COPD has been considered a self-inflicted disease caused by tobacco smoking and occurring primarily in older males^2^. This is a narrow and incomplete view, since COPD is similarly prevalent in men and women, and can be diagnosed in young individuals and even in never smokers^1^. In fact, GOLD 2023 proposes that COPD is actually the end-result of a series of dynamic, cumulative and repeated *gene* (G)–*environment* (E) interactions over the *lifetime* (T) that damage the lungs and alter their normal development/aging processes^3^. Below, we review the evidence supporting the influence of Genes, Environment and Time (*GETomics*) in the pathogenesis of COPD^3^.

### Genes

Mutations in SERPINA1 gene, leading to *α-1 antitrypsin deficiency* is the most relevant (albeit rare) genetic risk factor for COPD. Many other genetic variants have been recently identified as risk factors for reduced lung function and COPD, but their individual effect size is small^4^. The prevalence of COPD in males and females in developed countries is now very similar^5^ but some studies suggest more harmful effects of smoking among women^1,6^. For instance, females report more dyspnoea and cough, have a steeper decline in lung function over time and have worse outcomes than males in terms of hospitalizations, respiratory failure, and death^7^.

### Environment

*Cigarette smoking* is a key environmental risk factor for COPD; yet fewer than 50% of heavy smokers develop COPD^8^ and, as discussed below, about a third of patients with COPD have never smoked^9,10^. Passive smoking exposure also is a risk factor for COPD^11^. Smoking during pregnancy poses a risk for the *foetus*, by altering lung growth and development *in utero*^3,12^. In low- and middle-income countries (LMICs), *COPD in non-smokers* may be responsible for up to 60–70% of cases^10^. Wood, animal dung, crop residues, and coal (i.e., *biomass*), typically burned in poorly functioning stoves, may lead to very high levels of household air pollution^13^ and increase the risk for COPD. COPD in non-smokers is more common in females of younger age^1^. Symptoms and spirometric impairment are similar to those of smoking-induced COPD but emphysema is less prevalent and lung function decline less steep in non-smoking COPD. Research is needed to identify the most appropriate pharmacotherapy for this type of COPD^10^. *Occupational exposures*, including organic and inorganic dusts, chemical agents, and fumes^14,15^, and *air pollution* also increases the risk of COPD^16^.

### The time axis: lung function trajectories

**Fig. 1:**
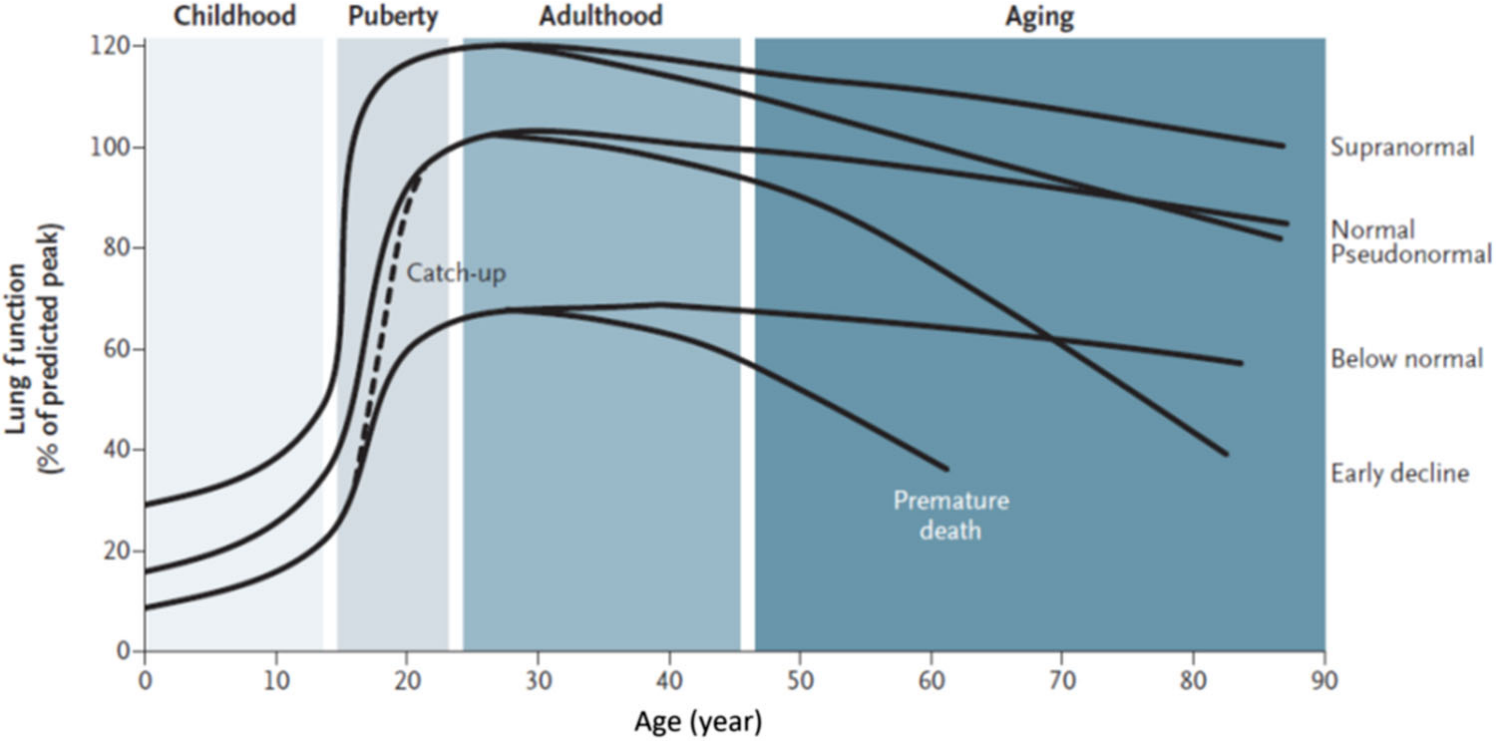
Range of lung function trajectories through the lifetime. For further explanations, see text. Reproduced with permission from reference ^89^.

At birth, the lungs are not fully developed. They grow and mature until about 20–25 years of age (earlier in females), when lung function reaches its peak^17^. This is followed by a relatively short *plateau* (which may vary from individual to individual) and a final phase of mild lung function decline due to physiological lung aging (Fig. 1). This normal *lung function trajectory* can be altered by processes occurring during gestation, birth, childhood, and adolescence that affect lung growth (hence, peak lung function) and/or processes shortening the *plateau* phase and/or accelerating the aging phase^18^ (Fig. 1). These processes include, among others, the following ones:

- *Childhood disadvantage factors*, such as prematurity, low birth weight, maternal smoking during pregnancy, repeated respiratory infections and poor nutrition are key determinants of peak lung function attained in early adulthood^19–26^. Reduced peak lung function in early adulthood increases the risk of COPD later in life^19,27,28^. In fact, approximately 50% of patients develop COPD due to accelerated decline in FEV_1_ over time while the other 50% develop it due to abnormal lung growth and development (with normal lung function decline over time)^29^.
- *Poverty and low socioeconomic status* increase the risk of COPD, likely because of exposure to household and outdoor air pollutants, crowding, poor nutrition, infections, or other factors related to low socioeconomic status^30^.
- Severe *respiratory infections* in childhood have been associated with reduced lung function and increased respiratory symptoms in adulthood^31,32^. In adults, chronic bronchial infection, particularly with *Pseudomonas aeruginosa*, is associated with accelerated FEV_1_ decline^33^. In many parts of the world, tuberculosis^34^ and HIV infection^35^ are also risk factors for COPD.

As a result of all these factors, in the general population there is a range of lung function trajectories through the lifetime^18^ (Fig. 1). Trajectories below the normal range are associated with a higher prevalence and earlier incidence of multi-morbidity and premature death^36^, whereas those above the normal range are associated with healthier aging, fewer cardiovascular and respiratory events, as well as with a survival benefit^37,38^.

## Taxonomy: beyond smoking

**Fig. 2:**
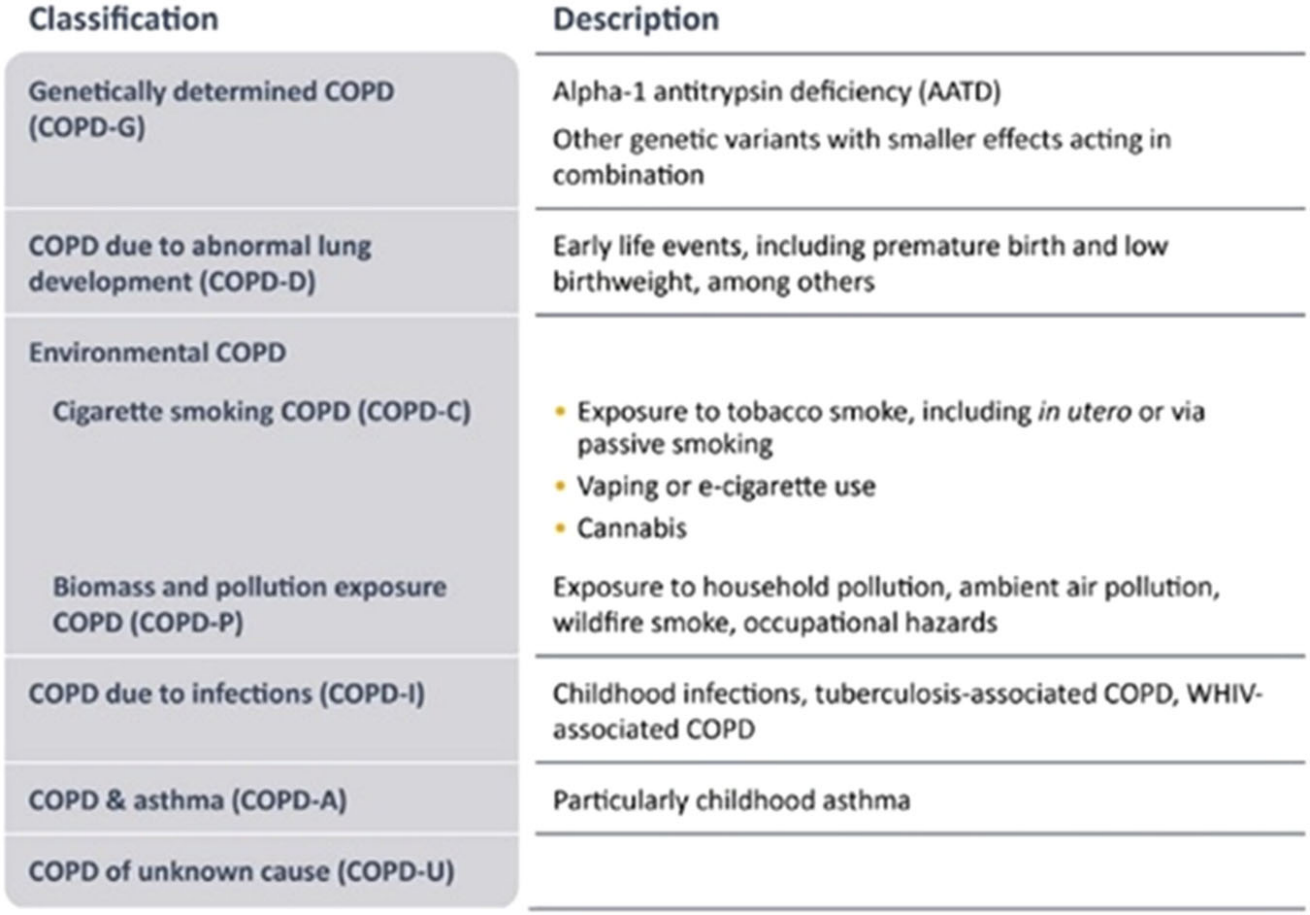
Proposed taxonomy (etiotypes) for COPD. Reproduced with permission from www.goldcopd.org.

Because it is now recognized that COPD can originate from multiples causes (*etiotypes*), GOLD 2023 proposes a new taxonomic classification (Fig. 2) that reflects two recent proposals^39,40^. This taxonomic classification does not yet have a direct clinical translation because scientific evidence on the natural history and/or best treatment of many of these etiotypes is still lacking (the vast majority of scientific evidence available relates to smoking-related COPD). However, it aims at raising awareness about these other, frequent, non-smoking related COPD and to stimulate research on the mechanisms, prevention, early diagnosis and management of these other etiotypes of COPD, which are highly prevalent around the globe^10^.

## Diagnosis: forced spirometry mandatory

A diagnosis of COPD should be *considered* in any patient who complains of dyspnoea, chronic cough or sputum production, a history of recurrent lower respiratory tract infections and/or a history of exposure to risk factors. However, forced spirometry showing the presence of a post-bronchodilator FEV_1_/FVC < 0.7 is *mandatory* to establish the diagnosis of COPD. There is a debate on whether it would be better to use the lower limit of normal of the FEV_1_/FVC ratio instead of a fixed value (<0.7). The full GOLD 2023 document (freely downloadable from www.goldcopd.org) discusses at length the pros and cons of both options. We invite the interested reader to read them there. In any case, it is of the outmost importance, thus, that all Primary Care Centres have access to standard spirometers. This should be considered a basic technological element included in the service portfolio of all public health centres. In addition, it is also essential to have professionals (physician, nurses, technicians) appropriately trained to perform valid spirometries. GOLD 2023 realizes, however, that this ideal scenario may not be feasible in LMIC^41,42^, but considers that it is important to state clearly that the diagnosis of COPD requires a spirometric measurement and that, without it, this diagnosis cannot be confirmed. Given the very large underdiagnosis of COPD, GOLD 2023 advocates active case finding (i.e., performing spirometry in patients with symptoms and/or risk factors), but not screening spirometry^1^. Small hand-held devices are useful to rule out COPD but not to confirm diagnosis.

Another important consideration here is that non-fully reversible airflow obstruction is *not specific for COPD* and can occur in other respiratory diseases (e.g., asthma, bronchiectasis, post-tuberculosis, etc.). Thus, it is very important that the clinical context and risk factors (see above) must also be considered when establishing a diagnosis of COPD.

The FEV_1_ values serve to determine the *severity of airflow obstruction* (GOLD grades 1,2,3, 4). The FEV1 thresholds for this severity gradation (mild (FEV1 ≥ 80% ref), moderate (FEV1 50-79% ref), severe (FEV1 30–49% ref) and very severe (FEV1 < 30% ref) have not changed from previous GOLD documents.

Finally, in asymptomatic individuals without any significant exposure to tobacco or other risk factors, *screening* spirometry is not indicated, but in those with symptoms and/or risk factors (e.g., >20 pack-years of smoking, recurrent chest infections, prematurity or other significant early life events), spirometry should be considered as a valid method for *case finding*^1^.

## pre-COPD and PRISm

GOLD 2023 also recognizes that some patients without airflow obstruction (i.e., FEV_1_/FVC > 0.7) may present symptoms and/or other functional abnormalities (e.g., reduced carbon monoxide diffusing capacity or enhanced rate of FEV_1_ decline) and/or structural lung abnormalities (e.g. emphysema on computed tomography (CT)) that may eventually progress (or not) to COPD (as defined by the presence of airflow obstruction); these patients are now termed pre-COPD^1^. Likewise, GOLD 2023 recognizes that there are patients with preserved FEV_1_/FVC ratio (so no evidence of airflow obstruction) with reduced FEV_1_; these patients are named PRISm (Preserved Ratio with Impaired Spirometry) and, like pre-COPD patients, may progress (or not) over time to COPD^1^. There is a lot to be learned about the mechanisms, natural history, and treatment of pre-COPD and PRISm patients, but the realization of their existence in real life open new opportunities for prevention, early diagnosis, and management^1^.

## Combined initial COPD assessment: from ABCD to ABE

**Fig. 3:**
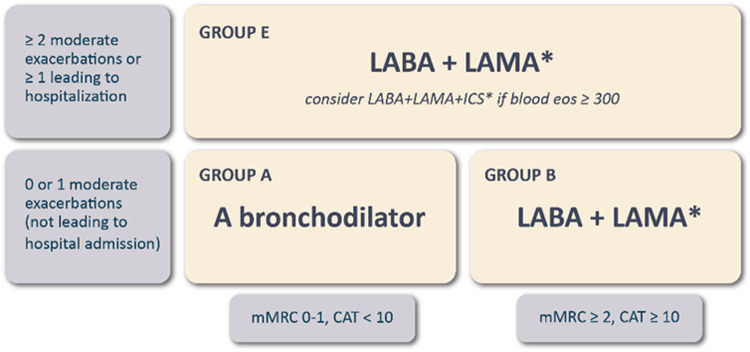
Initial pharmacological treatment. Exacerbation history refers to exacerbations suffered the previous year. *: single inhaler therapy may be more convenient and effective than multiple inhalers. mMRC: modified Medical Research Dyspnoea Questionnaire. CAT: COPD Assessment Test. LAMA: long-acting anti-muscarinic antagonist; LABA: long-acting β2 receptor agonist; ICS: inhaled corticosteroid; eos: eosinophils. Reproduced with permission from www.goldcopd.org.

GOLD 2023 modifies the previous ABCD assessment tool^43^ to a new one (ABE). This aims at recognizing the clinical impact of exacerbations, independently of the level of symptoms of the patient^44^ (Fig. 3). The thresholds proposed for symptoms (*X*-axis: mMRC or CAT above or below 1 or 10, respectively) and history of exacerbations in the previous year (*Y*-axis: 0-1 moderate exacerbations vs. ≥2 moderate exacerbations or ≥1 exacerbation leading to hospital admission) are unchanged from previous GOLD documents. In this 2023 proposal, therefore, the A and B groups remain unchanged, but the former C and D groups are now merged into a single group termed “E” (for “Exacerbations”). This has implications for the initial pharmacological treatment recommendations, as discussed below.

## Pharmacological treatment

### Choice and appropriate use of inhaler devices

Because inhaled therapy is the cornerstone of COPD treatment, the *appropriate use* of these devices (irrespective of the molecule(s) contained in them) is *essential* to optimize their therapeutic effect. This requires educating and training in the correct use of the device of both providers and patients: this is a key task of primary care professionals, preferably done in face-to-face consultations rather than by telemedicine. Regular assessment at follow-up is recommend to maintain their effective use regardless patients’ previous experience and time from first prescription. Patients’ and devices’ characteristics should be considered before making a decision about treatment. Box 1 summarizes the main principles that should be considered to guide the individualized selection of the appropriate device for a given patient^1^. Besides, the following aspects need to be considered also:

- If a patient is currently taking inhaled therapy and able to use their current device correctly, new therapy is best prescribed in the same device^1^. If a new device is required, either because the patient is not using their current device correctly or the drug is not available in the same device, an iterative process with the patient should be used to select a delivery system and ensure the patient can use it^1^.
- Appropriate education must be provided by health care professionals, including physical, video- or be-based demonstration of the proper technique and live verification that the patient masters this technique. It is crucial to check regularly (ideally, at each visit) that patients continue to use their devices correctly. The lack of placebo devices within clinical areas is often a limitation and barrier to providing quality inhaler technique instruction to patients. Encouraging a patient to bring their own devices to the clinic is a useful alternative.

Box 1: Basic principles for appropriate inhalation device choice (from reference ^1^)

- Availability of the drug in the device.
- Patients’ beliefs, satisfaction with current and previous devices and preferences need to be assessed and considered.
- The number of different device types should be minimized for each patient. Ideally, only one device type should be used.
- Device type should not be switched in the absence of clinical justification nor without proper information, education, and medical follow-up.
- Shared decision making is the most appropriate strategy for inhalation device choice.
- Patient’s cognition, dexterity and strength must be taken into account.
- Patient’s ability to perform the correct specific inhalation manoeuvre for the device must be assessed:
  - Dry powder inhalers (DPI) are appropriate only if the patient can make a forceful and deep inhalation. Check visually that the patient can inhale forcefully through the device - if there is doubt assess objectively or chose alternative device.
  - Metered-dose inhalers (MDI) and, to a lesser extent, slow mist inhalers (SMI) require coordination between device triggering and inhalation and patients need to be able to perform a slow and deep inhalation. Check visually that the patient can inhale slowly and deeply from the device - if there is doubt consider adding a spacer or chose alternative device.
  - For patients unable to use an MDI (with or without spacer), SMI or DPI a nebulizer should be considered.

- Other factors to consider include size, portability, cost.
- Smart inhalers may be useful if there are issues with adherence/persistence or inhalation technique (for devices that can check it)
- Physicians should prescribe only devices they (and the other members of the caring team) know how to use.

### *Initial* pharmacological treatment

As shown in Fig. 3, the recommended initial treatment of patients in Group A has not changed (a bronchodilator). In contrast, for patients in Group B, a dual long-acting bronchodilator combination (β2 adrenergic (LABA) + anti-muscarinic (LAMA) bronchodilators) is now recommended since dual therapy is more effective than monotherapy with similar side-effects^45–47^. The same initial treatment (LAMA + LABA) is also recommended for patients in group E, except for those individuals with blood eosinophils ≥ 300 cells/µL, in whom starting with triple therapy (LABA + LAMA + ICS) can be considered. The use of LABA + ICS in COPD is no longer encouraged^1^. If there is an indication for an ICS, then LABA + LAMA + ICS has been shown to be superior to LABA + ICS and is therefore the preferred choice^48,49^. If patients with COPD have concomitant asthma, they should be treated as if they have asthma^50^.

### ***Follow-up*pharmacological treatment**

**Fig. 4:**
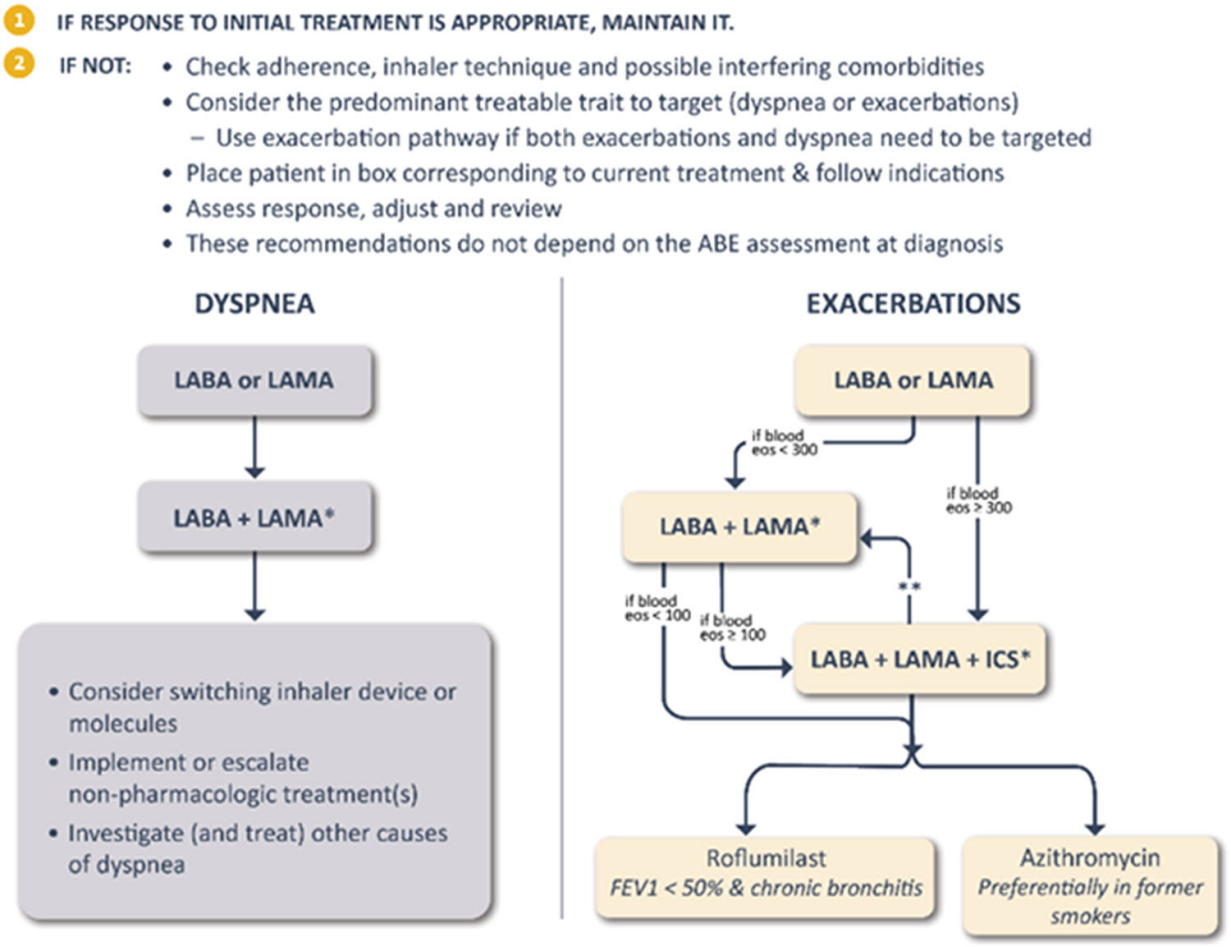
Follow-up pharmacological treatment. *: single inhaler therapy may be more convenient and effective than multiple inhalers; **: Consider de-escalation of ICS if pneumonia or other considerable side-effects. In case of blood eos ≥300 cells/μl de-escalation is more likely to be associated with the development of exacerbations. Exacerbation history refers to exacerbations suffered the previous year. mMRC: modified Medical Research Dyspnea Questionnaire. CAT: COPD Assessment Test. LAMA: long-acting anti-muscarinic antagonist; LABA: long-acting β2 receptor agonist; ICS: inhaled corticosteroid; eos: eosinophils. Reproduced with permission from www.goldcopd.org.

Following initiation of treatment, patients should be reassessed, and treatment should be adjusted if needed. GOLD 2023 continues to recommend that follow-up treatment be based on two key *treatable traits*^51,52^: dyspnoea and exacerbations (Fig. 4).

For patients with persistent *dyspnoea* on bronchodilator monotherapy (left column), it is critical to check inhaler technique; if good technique is assured, then a step up to LABA + LAMA is recommended if the patient was started on mono-bronchodilator treatment. If this does not improve symptoms clinicians should consider switching inhaler device or molecules, as well as investigating and treating other causes of dyspnoea and consider referral for pulmonary rehabilitation^1^.

For patients continuing to have *exacerbations* (with or without persistent dyspnoea) on bronchodilator monotherapy (right column), escalation to LABA + LAMA is recommended, except for patients with blood eosinophils ≥ 300 cells/µL who may be escalated to LABA + LAMA + ICS. For patients with persistent exacerbations on LABA + LAMA, escalation to LABA + LAMA + ICS is recommended if they have blood eosinophils ≥ 100 cells/µL. This is important since two recent large randomized clinical trials have shown that tiple therapy in patients with frequent exacerbations reduce all-cause mortality^53,54^. For patients continuing to exacerbate despite therapy with LABA + LAMA + ICS or those who have an eosinophil count of < 100 cells/µL, the addition of roflumilast (particularly in patients with chronic bronchitis and an FEV_1_ < 50% predicted)^55–57^ or a macrolide (particularly in patients who are not current smokers) may be considered^58,59^.

Patients whose pharmacological treatment has been modified should be closely monitored. ICS de-escalation or withdrawal can be considered if pneumonia or other considerable side effects occur, although if the blood eosinophil count is ≥300 cells/μl, ICS de-escalation is more likely to be associated with the development of exacerbations.

Finally, if a patient with COPD and no features of asthma has already been treated—for whatever reason—with LABA + ICS and is well controlled in terms of symptoms and exacerbations, then LABA + ICS could be continued. However, if they remain dyspnoeic switching to LABA + LAMA should be considered, and if they have further exacerbations, treatment should be escalated to LABA + LAMA + ICS.

## Non-pharmacological therapy

Non-pharmacological treatment is a *key* part of the adequate management of COPD and should always be considered in combination with the pharmacologic treatment discussed above. It includes one or more of the following^1^:

- *Education and supported self-management*. All patients should receive basic information about COPD and its treatment (respiratory medications and inhalation devices) and advice about when to seek help. Primary care is the right place to educate COPD patients and health-care professionals should be given the right tools to do that. However, education by itself does not often change behaviour. Education needs to be delivered in the context of a supportive behaviour change intervention that is personalized to the individual and their sociodemographic/cultural context.
- *Smoking cessation*. All patients who continue to smoke should be offered help and treatment to quit. Brief intervention in primary care is effective and pharmacologic treatment should be offered if possible^60^. Likewise, strategies for reducing exposure to indoor air pollution need to be considered too.
- *Vaccination*. Depending on local guidelines, patients should be offered vaccination against influenza, pneumococcus, COVlD-19, pertussis, and herpes zoster.
- *Physical activity*. All COPD patients should be encouraged to keep active. Technology-based interventions have the potential to provide convenient and accessible means to enhance exercise self-efficacy, and to educate and motivate patients to make healthy lifestyle changes^61^.
- *Nutritional and Psychosocial assessment and support* are important aspects to consider and treat if needed. Up to 50% of people with COPD weigh less than 90% of ideal body weight^62^. Dietary advice and oral supplementation have been reported to improve body weight, quality of life, respiratory muscle strength and 6-minute walk distance in patients with COPD^1^. Psychosocial consideration and support is also important in the management of these patients^1^.
- *Pulmonary Rehabilitation (PR)*. PR, including community and home-based, is beneficial^1^. Accordingly, patients with high symptom burden and risk of exacerbations (GOLD groups B and E) should be recommended to take part in a *formal PR program* designed and delivered in a structured manner, considering the individual’s COPD characteristics and comorbidities^63–66^. In some settings, this may be combined with rehabilitation for cardiovascular patients.
- *Oxygen therapy and ventilatory support*. The criteria for prescribing long term oxygen therapy and ventilator support remain unchanged and are described in detail in the GOLD 2023 report^1^.
- *Surgical and endoscopic lung volume reduction*. In selected patients with symptomatic heterogeneous or homogenous emphysema and significant hyperinflation refractory to optimized medical care, surgical or bronchoscopic modes of lung volume reduction may be indicated. The complete GOLD report provides specific recommendations for different procedures ^1^. Likewise, younger COPD patients with severe COPD should be considered for lung transplant^1^.
- *End of Life and Palliative Care*. All patients with advanced COPD should be considered for end of life and palliative care support to optimize symptom control and allow patients and their families to make informed choices about future management^1^.

## Exacerbations of COPD

### New definition

The previous GOLD definition of ECOPD was non-specific (“*acute worsening of respiratory symptoms that results in additional therapy*”) and its severity was determined *post facto* (mild, moderate or severe) based on the use of healthcare resources^67^. This is useless to guide treatment at the point of care.

To address these limitations, GOLD 2023 now proposes a more specific definition: “*ECOPD is an event characterized by increased dyspnoea and/or cough and sputum that worsens in* <*14 days which may be accompanied by tachypnoea and/or tachycardia and is often associated with increased local and systemic inflammation caused by infection, pollution, or other insults to the airways*”^68^. Providing a time frame (<14 days) facilitates the differentiation of an exacerbation of COPD from disease worsening. As discussed below, a number of biomarkers can help determining the severity of the ECOPD (hence, to guide treatment) at the point of care. Primary care is the most important setting for the detection and early recognition of signs and symptoms suggestive of an exacerbation of COPD.

### Differential Diagnosis

**Fig. 5:**
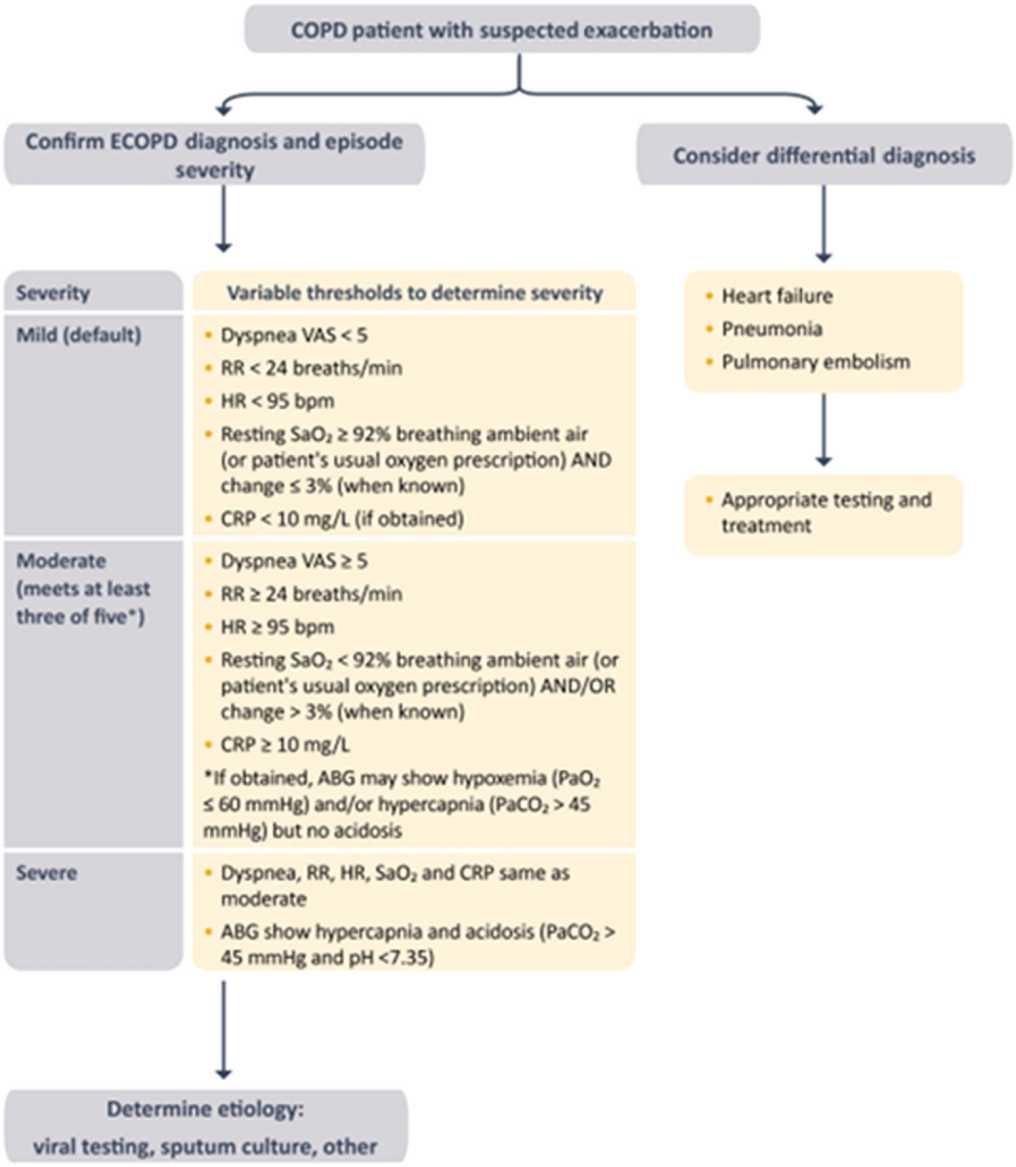
Classification of the severity of COPD exacerbations. Definition of abbreviations: VAS: visual analog scale; RR: respiratory rate; HR: heart rate; CRP: C-reactive protein. SaO_2_: arterial oxygen saturation; PaO_2_: arterial partial pressure of oxygen; ABG: arterial blood gases; ABG should show new onset/worsening hypercapnia or acidosis since a few patients may have chronic hypercapnia. Adapted from ref. ^68^. Reproduced with permission from www.goldcopd.org.

Patients with COPD are at increased risk of other acute events, particularly decompensated heart failure, pneumonia and/or pulmonary embolism that may *mimic* or *aggravate* an ECOPD (Fig. 5)^69^. Thus, careful differential diagnosis is essential since these other conditions also deserve treatment if present (Fig. 5).

### Assessment of ECOPD severity

GOLD 2023 suggests using several, easy to obtain clinical variables to define the severity of ECOPD (mild, moderate or severe) at the point of care (Fig. 5)^68^. In a primary care setting, severity can be determined by determining dyspnoea intensity (using a VAS 0 to 10 dyspnoea scale, with zero being not short of breath at all and 10 the worst shortness of breath you have ever experienced), respiratory rate, heart rate and oxygen saturation level; where available, measuring blood C-reactive protein (CRP) levels is recommended (Fig. 5). To move from a mild to a moderate level, three of the variables need to exceed the proposed thresholds (Fig. 5). To determine the need for ventilatory support (usually in the emergency room or hospital setting) arterial blood gases should be measured.

### Management of ECOPD

#### Treatment setting

Depending on the episode severity, as well as that of the underlying COPD and comorbidities, an ECOPD can be managed in either the outpatient or inpatient setting. The following are *indications for hospitalizati*on: (1) severe symptoms such as sudden worsening of resting dyspnoea, high respiratory rate, oxygen saturation ≤92%, confusion, drowsiness; (2) acute respiratory failure; (3) onset of new physical signs (e.g., cyanosis, peripheral oedema); (4) failure to respond to initial medical management; (5) presence of serious comorbidities (e.g., heart failure, newly occurring arrhythmias, etc.); and, (6) insufficient home support^1^.

#### Pharmacological treatment

- *Bronchodilators*. Short-acting inhaled β2-agonists (SABA), with or without short-acting anticholinergics (SAMA), are the initial bronchodilators for acute treatment of ECOPD, administered using a metered-dose inhaler (MDI, with a spacer device if necessary, or nebulization^1^. If a nebulizer is chosen, air-driven is preferable to oxygen-driven nebulization to avoid the potential risk of increasing PaCO_2_^70^. Intravenous methylxanthines (theophylline or aminophylline) are not recommended due to lack of efficacy and significant side effects^71,72^.
- *Glucocorticoids*. Systemic glucocorticoids in COPD exacerbations improve lung function, oxygenation, risk of early relapse, and reduce treatment failures and length of hospitalization^73–75^. A dose of 40 mg prednisone-equivalent per day for 5 days is recommended^76^. Longer courses increase risk of pneumonia and mortality^77^. Therapy with oral prednisolone is equally effective as intravenous administration^78^.
- *Antibiotics*. Antibiotics should be given to patients with ECOPD who have increased sputum volume and sputum purulence and most of those require mechanical ventilation (invasive or non-invasive)^79^. CRP-guided prescribing of antibiotics for ECOPD in primary care clinics resulted in a reduced proportion antibiotic use with no evidence of harm^80^. The recommended length of antibiotic therapy is 5-7 days^81^. The choice of the antibiotic should be based on the local bacterial resistance pattern.

#### Non-pharmacologic treatment

- *Oxygen therapy*. Supplemental oxygen for hypoxemia should be titrated to a target saturation of 88-92%^82^. Venturi masks offer more accurate and controlled delivery of inspired oxygen than do nasal prongs^1^.
- *Non-invasive ventilatory support (NIV)*. NIV is indicated in patients with respiratory acidosis since it improves gas exchange and decreases respiratory rate, work of breathing, the severity of breathlessness, intubation rates, and mortality^83,84^.

It is important that patients are reviewed clinically, and treatment adjusted if needed, after the exacerbation episode.

## Comorbidities, multimorbidity and frailty

COPD almost invariably coexists with other chronic diseases (multimorbidity) that affect the patient’s clinical condition^85^. In general, the presence of comorbidities should not alter COPD treatment and comorbidities should be treated per usual standards regardless of the presence of COPD^1^. The most common comorbidities include:

- *Cardiovascular diseases*, including hypertension, ischemic heart disease, congestive heart failure, arrythmias, and peripheral vascular disease.
- *Lung cancer*. Primary care professionals should always keep in mind that former or current smokers with COPD, particularly those with emphysema, are at higher risk of lung cancer. Given that lung cancer screening with low-dose CT is now recommended in many countries because it reduces all-cause mortality in older current or former smokers in the general population (https://view-health-screening-recommendations.service.gov.uk/lung-cancer/) it may seem advisable to consider early lung cancer detection by CT-scan in COPD patients seen in primary care.
- *Bronchiectasis*. A chest CT scan is recommended if bronchiectasis is suspected.
- *Sleep apnoea* occurs in about 14% of COPD patients and worsens their prognosis.
- *Osteoporosis*. Osteoporosis is often under-diagnosed and associated with poor health status and prognosis. Recurrent use of systemic corticosteroids increases the risk of osteoporosis and should be avoided if possible.
- *Diabetes and metabolic syndrome*. Both are frequent in COPD and affect their prognosis.
- *Gastroesophageal reflux*. It is an independent risk factor for exacerbations and is associated with worse health status.
- *Anaemia and polycythaemia* can occur in patients with COPD and impact their health status and prognosis.
- *Mental health*. Anxiety and depression are important and underdiagnosed comorbidities in COPD.

## Copd and Covid-19

COPD patients are not at increased risk of infection with SARS-CoV-2 but, if they get infected, then they do have a higher risk of hospitalization, ICU admission, and mortality^86^. Thus, COPD patients should follow strictly preventive measures, including social distancing and washing hands, wearing a facial mask, should receive COVID-19 vaccination in line with national guidelines and should keep taking their oral and inhaled respiratory medications for COPD ^1^. Patients should stay in contact with their friends and families by telecommunication and continue to keep active.

## Conclusions

COPD is a common, preventable, and treatable disease, but extensive under-diagnosis and misdiagnosis leads to patients receiving no treatment or incorrect treatment^1^. The realization that environmental factors other than tobacco smoking can contribute to COPD, that it can start early in life and affect young individuals, and that there are precursor conditions (“Pre-COPD”, “PRISm”), opens new windows of opportunity for its prevention, early diagnosis, and prompt and appropriate therapeutic intervention^40,87^. Importantly, several pharmacological (triple therapy) and non-pharmacological therapies (smoking cessation, long-term oxygen therapy, non-invasive positive pressure ventilation and lung volume reduction surgery) have now been shown to reduce mortality of COPD patients^1^ but, in order to implement them, COPD must be first diagnosed. Thus, any strategy aimed at addressing and improving the huge underdiagnosis of COPD in the community should be reinforced. This is particularly relevant in a Primary Care setting. Further, because spirometry may not only diagnose respiratory diseases, but it can also identify a group of young adults (20-25 years of age) at risk of other cardiovascular and metabolic comorbidities and premature mortality^36^, it has been proposed as a global marker of health^88^. Box 2 summarizes the main recommendations for the pharmacologic treatment of COPD in Primary Care.

Box 2. Key headlines for GOLD pharmacological treatment recommendations of COPD (www.goldcopd.org)

- For symptomatic patients, a LABA-LAMA therapy in a single inhaler is recommended as initial therapy.
- The combination of LABA-ICS is no longer recommended in patients with COPD.
- Triple therapy (LABA-LAMA-ICS) is recommended in COPD patients who still suffer exacerbations of the disease despite LABA-LAMA therapy, if blood Eosinophil levels are higher than 100 cells/μl.
- ICS are not recommended in patients with <100 Eos/μL.
- Pharmacologic tratement must always be combined with non-pharmacologic treatment (including adequate treatment compliance, smoking cessation, physical activity and appropriate vaccination), and consideration of coexistent comorbidities.

### Reporting summary

Further information on research design is available in the Nature Research Reporting Summary linked to this article.

## Supplementary Information

Reporting summary

## Data Availability

This manuscript summarizes the GOLD 2023 recommendations for the diagnosis and management of patients with chronic obstructive pulmonary disease (COPD). The full document can be downloaded from www.goldcopd.org.
